# A Systematic Review of the Current Role of Minimally Invasive Spine Surgery in the Management of Metastatic Spine Disease

**DOI:** 10.1155/2011/598148

**Published:** 2011-06-02

**Authors:** Camilo A. Molina, Ziya L. Gokaslan, Daniel M. Sciubba

**Affiliations:** Department of Neurosurgery, Johns Hopkins University School of Medicine, 600 N. Wolfe Street, Meyer Building 5-185a, Baltimore, MD 21287, USA

## Abstract

Although increasingly aggressive decompression and resection methods have resulted in improved outcomes for patients with metastatic spine disease, these aggressive surgeries are not feasible for patients with numerous comorbid conditions. Such patients stand to benefit from management via minimally invasive spine surgery (MIS), given its association with decreased perioperative morbidity. We performed a systematic review of literature with the goal of evaluating the clinical efficacy and safety of MIS in the setting of metastatic spine disease. Results suggest that MIS is an efficacious means of achieving neurological improvement and alleviating pain. In addition, data suggests that MIS offers decreased blood loss, operative time, and complication rates in comparison to standard open spine surgery. However, due to the paucity of studies and low class of available evidence, the ability to draw comprehensive conclusions is limited. Future investigations should be conducted comparing standard surgery versus MIS in a prospective fashion.

## 1. Introduction

It is estimated that nearly 10 million people worldwide were diagnosed with cancer in 2000, with the incidence expected to increase to 15 million by 2020 [1]. The most commonly diagnosed neoplasms are breast, lung, and prostate cancers [2, 3]. Metastatic invasion of the spinal column can occur via various mechanisms that are dependent on both the biological behavior and physical location of the primary tumor [4]. Given the predilection of the breast, prostate, and lung neoplasms to metastasize to bone, it is not surprising that spinal metastases occur in 30–90% of patients, with 10% of such patients experiencing symptomatic metastatic epidural spinal cord compression (MESCC) [4, 5]. The most common symptom at presentation is pain that can be both radicular (exaggerated by percussion or palpation) and/or mechanical (exacerbated by movement) [6, 7]. Neurological dysfunction including motor, sensory, and autonomic dysfunction is the second most common presentation modality and is indicative of metastatic epidural spinal cord compression (MESCC) [3, 8–10]. 

Ideal management is multidisciplinary and involves various medical specialties such as neurosurgery, surgical oncology, medical oncology, radiation oncology, interventional radiology, pain specialists, and rehabilitation therapy [4, 5]. Management strategies involve a combination of surgery (for candidate patients), radiotherapy, and pharmacotherapy [4, 5, 11]. Due to both the short life expectancy of afflicted patients and high systemic tumor burden [8, 9, 12–14], with the exception of solitary metastatic lesions such as in the setting of renal cell carcinoma, treatment regimens are most often palliative rather than curative [10]. Afflicted patients frequently present with infiltration of the spinal column with tissues that lack weight bearing properties resulting in spinal instability, particularly ventral column instability given that most metastatic lesions localize to the anterior elements [11]. Optimal treatment of such patients requires stabilization in addition to traditional (surgical or nonsurgical) decompression [4, 15]. The most efficacious modality for restoring column instability is reconstructive surgical intervention. Unfortunately, numerous patients are not considered candidates for surgical intervention due to neoplasm-associated comorbidities such as malnourishment and diminished immune system that make extensive surgical procedures unfeasible [4]. Such patients can be managed with vertebral augmentation, as it can provide some degree of restabilization [11]. However, surgical advances in the field of minimally invasive spine surgery (MIS) have opened the door for not only extended surgical candidacy to patients who were previously ineligible, but it has also established the setting for surgical intervention with minimal perioperative morbidity such as decreased pain, less blood loss, and shorter hospital stays [11, 15–23]. This paper aims to describe the current role of MIS in the treatment of metastatic spine disease. The overall objectives of this paper are to present a systematic review of literature with regard to the following clinical questions:

1. the efficacy of MIS in improving neurological and pain-associated outcomes in the setting of metastatic spine disease;
2. the incidence of complications associated with MIS in the setting of metastatic spine disease.

## 2. Methods

### 2.1. Search Strategy

A systematic review of literature was performed employing Pubmed and a review of bibliographies of reviewed articles. The search query was broad and formulated to combine a number of subheadings and keywords that included the therapies and pathology of interest. The search string employed was the following:

 (“Minimally Invasive Surgery” OR “MIS” OR “VAST” OR “endoscopic thoracoscopy” OR “mini-open spine surgery” OR “minimal access spine surgery” OR “MASS”) AND ((“bone neoplasms” (Mesh) OR “spinal neoplasms” (Mesh)) OR (“spin*” AND “metasta*”) OR (“Spinal Cord Compression” (Mesh) OR “spinal cord compression”) OR (“epidural neoplasms” (Mesh) OR “epidural neoplasm”)). 

### 2.2. Eligibility Criteria

- Criteria for possible inclusion were the following:
  - articles published between 1980 and 2011,
  - all articles in English or with an English translation,
  - adult age group (18 years and older),
  - articles describing the use of minimally invasive spine surgery modalities in the treatment of metastatic disease,
  - fully published peer reviewed studies including RCTs, nonrandomized trials, cohort studies, case control studies, case series, and case reports. Both prospective and retrospective studies were considered.

(ii) Criteria for exclusion were the following:
  - iontradural spine tumors,
  - primary spine tumors,
  - pediatric age groups,
  - articles with no extractable data specific to metastatic spine disease.

### 2.3. Study Eligibility and Quality Assessment

Abstracts were screened by two independent reviewers using the above-stated inclusion and exclusion criteria. Cases of reviewer disagreement were resolved by a third reviewer. Full-text versions of acceptable article were gathered and subjected to more detailed screening for inclusion. After finalizing a collection of eligible studies, the studies were analyzed in detail, and the data pertaining to the research questions was extracted and tabulated by one reviewer. The second reviewer checked the extracted information. 

## 3. Results

A total of eleven publications were ultimately found eligible to evaluate the clinical outcomes associated with MIS as a treatment for metastatic spine disease. All of the publications available were retrospective in nature. Nine of the publications were retrospective case series, and two of the publications were case reports. Although case reports are normally excluded in systematic reviews, they were included in this review due to the paucity of evidence evaluating MIS in the setting of metastatic spine disease. The main outcomes extracted from the selected publications included mean operating time (MOT), mean blood loss (MBL), hospital length of stay (LOS), rate of neurological improvement (NI), pain alleviation rate (PA), and complication rate (CR). Collected outcomes are tabulated in Tables 1 and 2.

### 3.1. Video-Assisted Thoracostomy (VAST)

There were a total of five publications addressing the use of VAST or endoscopy-assisted posterior decompression to manage patients with metastatic spine lesions. Four of the publications were retrospective case series, and one was a case report. The earliest description of VAST for managing metastatic vertebral was published by Rosenthal et al. [20] in 1996. The authors described the development of an endoscopic procedure to achieve anterior vertebrectomy, reconstruction, and stabilization of the thoracic spine in 4 patients afflicted with metastatic spine lesions. All patients were in good health condition but were experiencing progressive neurological decline and radiological evidence of bone destruction and cord compression. The study reported a 6.5 hr MOT, 7.5 day LOS, and 1450 mL MBL. The authors found that MBL was correlated to MOT and extent of vertebrectomy. Additionally, all of the patients were ambulatory with assistance on postoperative day 1, ambulatory with a Jewett brace during the first 4 weeks, and independently ambulatory at 11-month followup (NI: 100%). Patients were pain-free following chest drain removal on day 3 or 4 and remained pain-free at 11-month followup (PA: 100%). The study reported no complications (Table 1). 

Huang et al. [24] published a retrospective case review of 90 patients who had undergone VAST for various spinal pathologies, of which 41 cases were due to metastatic lesions. The main goal of the study was to evaluate MIS complication rates. Procedures performed for the metastatic lesion afflicted subgroup included biopsy only, corpectomy for decompression, and corpectomy with interbody fusion. Although the study did not stratify MOT (3.1 h) or MBL (775 mL) according to neoplastic or nonneoplastic etiologies, the study did stratify complication rates. The authors reported a total of 30 complications in 22 patients (overall CR: 33%) for the 90 procedures performed. Importantly, 22 of those complications occurred among the 41 patients treated for metastatic spine disease (CR: 54%). Additionally, the authors also noted that the most common complication was excessive intraoperative bleeding, with all 5 instances occurring in patients with metastatic disease. The additional complications encountered were intercostals neuralgia (7%), superficial wound infection (7%), atelectasis (5%), pericardial penetration (2%), implant failure (2%), and death (2%). Notably, none of the complications occurred due to injury to the spinal cord, a great vessel, or internal organ (Table 1). 

Le Huec et al. [25] published a small case series of two patients in which VAST was used to manage metastatic spine disease encompassing the cervicothoracic junction. The goal of the authors was to develop an alternative approach to the traditional lateral approach that requires mobilization of the scapula to visualize the T1, T2, and T3 spinal levels. The technique was technically feasible and allowed for ample access to achieve corpectomy and visualization of the posterior longitudinal ligament, thereby allowing for complete release of the cord. MOT was 2.6 hours, MBL was 350 mL, and mean LOS was 6.5 days. Both patients presented with progressive neurological decline but were independently ambulating at last followup (7 and 12 months) (NI: 100%). Both patients experienced substantial pain relief (PA: 100%), but one required narcotics at the followup due to having undergone additional surgeries for other metastases. One patient acquired a progressive recurrent laryngeal nerve palsy (CR: 50%) (Table 1). 

### 3.2. Endoscopy-Assisted Posterior Decompression

McLain [21] reported a retrospective case series of 8 patients afflicted with metastatic spine lesions to demonstrate the feasibility of endoscopically assisted (transpedicular) decompression and stabilization through a single, extrapleural, and posterolateral approach. MOT was 6.5 hours, and MBL was 1677 mL. All 6 of the patients that presented with neurological deficit recovered completely and maintained neurological integrity until the last followup or terminal care (3–36 months) (NI: 100%). The other 2 patients not presenting with neurologic compromise retained neurological function until the last followup or terminal care (3–36 months). All 8 patients experienced pain relief (PA: 100%), and 5 patients (62.5%) did not require any analgesics at the last followup. The authors concluded that endoscopy augmented the efficacy of the posterolateral approach by improving the visualization of structures that were traditionally difficult to access through a standard posterolateral approach (Table 1). 

Mobbs et al. [26] published a case report of endoscope-assisted posterior decompression of a solitary renal cell carcinoma metastatic lesion. The patient initially presented with hyperreflexia and back pain but was neurologically intact and pain-free at two-month postoperative followup. The patient's course was uncomplicated throughout the procedure and postoperative recovery (Table 1). 

### 3.3. Minimal Access Spine Surgery (MASS)

There were a total of six publications addressing the use of MASS to manage patients with metastatic spine lesions. Muhlbauer et al. [27] published the first description of MASS for managing metastatic spine disease in 2000. The authors reported a small retrospective case series regarding the management of 5 patients with compression fractures from osteoporosis or metastatic lesions. Reported MOT was 6 hours, and MBL was 1120 mL. All 5 of the patients presented preoperatively with both pain and neurological dysfunction. At followup, all patients had experienced neurological improvement (NI: 100%) characterized by either progressing from ambulating with a cane to ambulating unassisted, or from being nonambulatory to ambulating with a cane. Additionally, all patients experienced significant pain relief (PA: 100%) with 40% of the patients not utilizing analgesics at followup (6–12 months) (Table 2).

Huang et al. [23] published a retrospective analysis of 46 patients to compare outcomes in MASS (*n* = 29) and standard thoracotomy (ST, *n* = 17) in the setting of metastatic spine disease. There was no significant difference in MOT, MBL, NI, or CR. MOT for MASS was 179 minutes versus 180 minutes for ST (*P* = .54). MBL for MASS was 1,100 mL versus 1,162 mL for ST (*P* = .63). Neurological outcome was reported as the postoperative reacquisition of ambulation. NI for MASS was 70.8% versus 69.2% for ST (*P* = .6). CR for MASS was 24% versus 29% for ST (*P* > .05). Complications encountered from MASS included dural tears (2), femoral fracture (1), pneumothorax (1), tumor recurrence (1), implant failure (1), and metastasis (1). Complications encountered from ST included sepsis (1), postoperative pneumonia (1), pneumothorax (1), GI bleeding (1), and UTI (1). Additionally, 2 year survival rates were also not significantly different (MASS: 24% versus ST: 29%, *P* = .69). However, the authors found that the percentage of patients requiring at least a 2-day postoperative admission to the intensive care unit (ICU) was significantly different when comparing MASS to ST, with MASS resulting in significantly less admissions (MASS 6.9% versus ST: 88%, *P* ≤ .001) (Table 2). 

Deutsch et al. [28] reported a retrospective case series of 8 patients undergoing MASS posterolateral vertebrectomy and decompression to treat symptomatic thoracic MESCC. The patient population was compromised of patients not deemed candidate for conventional open thoracotomy due to age (mean 74 y), limited life expectancy, and/or systemic metastatic burden. MOT was 2.2 hours and MBL was 227 mL. All patients presented with substantial neurologic deficit (mean Nurick grade: 4.35 (range 3–5)) and pain (mean numerical pain score (NPS) 5.5 (range 3–8)). Postoperatively, 5 patients experienced neurologic improvement (NI: 62.5 %), and the mean Nurick grade of all patients decreased to 3.13. 5 patients experienced pain alleviation (PA: 62.5%), with the group mean NPS decreasing to 3.10. There was no incidence of complications reported (Table 2). 

Kan and Schmidt [29] published a retrospective case series of 5 patients with metastatic disease of the thoracic spine who underwent ventral decompression via MASS. The procedure included a corpectomy, interbody fusion, expandable cage-mediated reconstruction, and stabilization via anterior plating through MASS techniques. MOT was 4.3 hours, MBL was 610 mL, and mean LOS was 6.25 days. All patients who presented with neurological deficits were neurologically intact at 6-month followup (NI: 100%). The preoperating mean VAS score for the group was 6.8, and it decreased to 3 at 6-month followup. Additionally, all patients experienced some degree of pain relief (PA: 100%) (Table 2).

Payer and Sottas [30] published a case series of 37 patients, 11 of which were afflicted with thoracic metastasis to the spine and managed via MASS using the SynFrame (Stratec Medical; Obendorf, Switzerland) table mounted retractor. The authors stratified results according to tumor and nontumor etiology. MOT for tumor patients was 188 minutes versus 178 minutes for nontumor patients. MBL for tumor patients was 711 mL versus 598 mL for nontumor patients. There were 4 complications (15%) in the nontumor group and 2 complications in the tumor group (18%). Neurological outcomes were not stratified according to etiology. However, it was reported that of the 22 patients presenting with neurological deficits, 20 patients demonstrated recovery (NI: 92%). Preoperative and postoperative pain outcomes were not compared (Table 2).

Taghva et al. [31] published a case report describing a T4 and T5 vertebrectomy with expandable cage placement coupled with T1–T8 screw fixation and fusion using MASS. The patient was afflicted with metastatic adenocarcinoma of the lung and presented with back pain for more than 4 months. On neurological examination, the patient was found to have decreased strength and sensation. Operative time was 7 hours, and blood loss was 1200 mL. The patient was discharged 5 days following surgery. Neurological outcome was positive, with the patient being ambulatory postoperatively on day 1 and completely recovering strength and sensory function at 9-month followup. Similarly, pain alleviation was satisfactory with the patient reported to be pain-free at 9-month followup (Table 2). 

### 3.4. Summary

There were a total of 5 publications, encompassing a total of 105 patients, selected to review the outcomes of VAST and endoscopy-assisted posterior decompression in the setting of metastatic spine disease. Data was compiled and yielded a median MOT of 4.6 hours (2.6–6.5 hours), a median MBL of 1113 mL (350–1677 mL), 7-day median LOS (6.5–7.5 days), 100% median NI (92%–100%), 100% median PA (94%–100%), and 0% median CR (0%–54%) (Table 3) Data gathered from the 6 publications, totaling 76 patients, to evaluate MASS outcomes in the setting of metastatic spine disease yielded similar results with a median MOT of 3.7 hours (2.2–7 hours), a median MBL of 905 mL (227–1200 mL), 5-day median LOS (4–6.25 days), 95% median NI (62.5%–100%), 100% median PA (62.5%–100%), and 9% median CR (0%–24%) (Table 3). In comparing VAST to MASS (Table 3), the data suggests that VAST was associated with longer operative times, increased hospital length of stay, and increased blood loss. However, VAST compared favorably when looking at median neurological improvement and median complication rates. Despite appearing clinically significant, it is uncertain whether these differences are statistically significant.

## 4. Discussion

Surgical intervention in the setting of metastatic spine disease commenced prior to the advent of radiotherapy, and the initial goals of treatment were to achieve decompression of the spinal cord. This was most commonly performed via a dorsal laminectomy, as it was believed that this would relieve the pressure on the cord resulting in a reversal of neurologic deficits. However, the majority of metastatic neoplasms affect the anterior column and thus when combined with destabilization of the posterior column via a laminectomy, patients experienced rapid destabilization of the entire spinal column along with both cord vascular insufficiency and radicular compression due to the loss of spinal column integrity [2, 32]. 

With the advent of radiotherapy, evidence accrued demonstrating no neurological benefit to surgical intervention, specifically laminectomy alone, in comparison to radiotherapy alone, and thus surgery as a primary treatment modality was abandoned [33–36]. However, spine surgery in the setting of the metastatic spine disease continued to advance as surgeons continued to operate in patients whose neurological function was not improved following radiotherapy [11]. During the 1980s, rapid advances in both surgical technique and advances in spinal instrumentation resulted in the publication of the studies that re-established a role for surgical intervention as an addition to radiotherapy [37, 38]. In 1983, Constans et al. [39] published a retrospective case series of 600 patients with symptomatic MESCC and reported a neurological stabilization rate of 41% and a neurological improvement rate of 44%, both of which were rates considered to be superior to prior reported rates. In 2004, Klimo Jr. et al. [40] published a meta-analysis comparing outcomes of surgery and radiotherapy management compared to radiotherapy alone and reported superior outcomes for patients who underwent surgery in addition to radiotherapy. In 2005, Patchell et al. [41] conducted the first randomized control study comparing the efficacy of radiotherapy and surgery to that of radiotherapy alone. Similar to the results of Klimo Jr. et al. [40], the study not only found functional and survival outcomes to be superior in the surgery plus radiotherapy group but also reported that surgical intervention was cost effective, cementing the role of surgery in the management of metastatic spine disease for candidate patients.

Although surgery plus radiation has been shown to be superior to radiation alone in a class I study, the role of surgical intervention remains controversial due to the difficulty of appropriate patient selection. Numerous factors such as tumor type, extent of metastatic disease, spinal stability, neurologic status, comorbid conditions, and life expectancy are considered when evaluating a patient for potential surgical candidacy [4, 15]. Furthermore, numerous scoring systems such as that of Tokuhashi et al. [42] and Tomita et al. [38] have been created to guide patient selection and dictate the aggressiveness of the respective surgical intervention. Unfortunately, the advances in surgical technique that improved surgical outcomes in patients with metastatic lesions required aggressive methods such as circumferential decompression or combined (anterior, posterior, and lateral) approaches that were only feasible in healthier patients with respective longer life expectancies and thus were not feasible for patients with numerous comorbid conditions or contraindications such as ongoing chemotherapy [15]. 

Minimally invasive spine surgery was created with the purpose of minimizing soft tissue surgical trauma and thereby accelerating postoperative care [16, 18, 43, 44], without a loss of surgical effectiveness, and was thus applicable to the management of metastatic spine disease in patients not candidate for conventional surgical intervention. More specifically, patients with single or adjacent level involvement with neurologic symptoms from spinal instability or neurological structure compression and a life expectancy of at least 3 months are considered candidate for MIS [15, 16, 18]. 

There are two main modalities of minimally invasive spine surgery: endoscopic video-assisted thoracoscopic surgery (VAST) and mini-open surgeries otherwise known as minimal access spine surgery (MASS) [15]. VAST, first described in 1993 [45], allows for the visualization and magnification of the entire ventral spine from T1 to T12, thereby allowing for decompression, reconstruction, and stabilization similar to an open thoracotomy. However, unlike an open thoracotomy, VAST has the advantage of decreased pulmonary morbidity, preservation of chest wall motion, decreased intercostal neuralgia, and avoidance of scapular dysfunction [46]. Furthermore, VAST can be combined with laparoscopic techniques to permit similar visualization and manipulation of the lumbar spine [15, 29]. Despite advantages, VAST has not become a widely adopted procedure due to practical limitations such as a steep learning curve, increased surgical time, relative difficulty in controlling intraoperative bleeding, and expensive equipment needed to perform the procedure [15, 47]. 

MASS was first described in 1997 [48] as a microsurgical approach for performing an anterior lumbar fusion, covering all levels from L2 to S1. It has since become more popular than VAST as an MIS modality as it is easier to learn, is a more familiar exposure to most spine surgeons, permits faster decompression of the spinal canal [23, 30, 49], potentially allows for safer mobilization of neurovascular structures, and provides three-dimensional direct vision allowing for easier reconstruction of the anterior column [50]. Since its introduction, the procedure has been modified to permit access from T2 to S1 via a combination of mini-open thoracotomy and/or retroperitoneal miniapproach [30, 49]. 

In this study, we performed a systematic review of published literature to date with the goal of evaluating the clinical efficacy and safety of MIS in the setting of metastatic spine disease. A total of 11 studies specifically reporting outcomes of metastatic spine cases managed via MIS were gathered. 5 of the studies, totaling 105 patients employed VAST, and 6 of the studies, totaling 76 patients, employed MASS. All of the collected studies were retrospective (Class IV evidence), and two of the studies were case reports. Although traditionally excluded from systematic reviews, the two case reports collected were included in our study due to the scarcity of published studies reporting on the use of MIS to treat metastatic spine lesions. 

We evaluated the clinical efficacy of MIS for the treatment of metastatic vertebral lesions via neurological improvement rate and pain alleviation rate outcome data. Collected data from each study was compiled to yield median mean neurological improvement (mNI) and median mean pain alleviation rate (mPA). mNI for VAST was 100% (92%–100%) and 95% (62.5%–100%) for MASS. mPA for VAST was 100% (94%–100%) and 100% (62.5%–100%) for MASS. The neurological improvement and pain alleviation rates are similar to those provided by the Class I study conducted by Patchell et al. that evaluated surgery plus radiotherapy outcomes [41]. Given the high rates of neurological dysfunction and pain alleviation, the results suggest that both VAST and MASS are efficacious means of achieving pain and neurological dysfunction relief through decompression and stabilization. 

Operative variables such as operative time, blood loss, complication rate, and hospital stay are considered markers of safety and practicality. Prolonged operating times are associated with an increased amount of complications (i.e., higher wound infection rate) and costs [51]. High blood loss leading to perioperative anemia leads to increased morbidity (i.e., surgical site infections), mortality, length of stay, and readmission rates [52]. Furthermore, patients with high blood loss often require transfusions which are associated with higher risks of infection, acute immune-mediated hemolytic reactions, and gastrointestinal complaints [52]. Longer hospital stays result in higher costs and are indicative of increased patient morbidity [15]. Smith et al. [47] compiled median operative variables of 16 studies, totaling 746 patients, reporting outcome data for open thoracotomies performed in the setting of thoracolombar spine pathology. One of the limitations commonly associated with MIS procedures is prolonged operative time. Data gathered contradicts this notion and suggests that both VAST and MASS collective median operating times (mMOT) compare favorably to open standard thoracotomy (ST) operating times collected by Smith et al. [47] (VAST: 4.6 hours (2.6–6.5 hours); MASS: 3.7 hours (2.2–7 hours); ST: 4.65 hours (2.3–10.2 hours)). 

Decreased complication rates, blood loss, and length of stay are considered to be among the benefits of MIS. This was confirmed by outcomes data compiled in our study when compared to gathered data outcomes for ST [47]. Median mean complication rates (mCR) for VAST (0% (0%–54%)) and MASS (9% (0%–24%)) compared favorably to those of ST (30.5% (15%–94.4%)). Similarly, median mean blood loss (mMBL) and median mean length of stay (mLOS) for both VAST (mMBL: 1113 mL (350–1677 mL); mLOS: 7 days (6.5–7.5 days)) and MASS (mMBL: 905 mL (227–1200 mL); mLOS: 5 days (4–6.25 days)) was decreased in comparison to data gathered for ST [47] (mMBL: 2100 mL (460–3136 mL); mLOS: 14.6 days (7.2–35.5 days)). It should be noted that the paper by Huang et al. [23] included in this review performed a direct retrospective comparison of MOT, MBL, LOS, and CR for MASS versus ST and found no significant difference in rates for any of the latter. However, the study did find a significant difference in the incidence of patients that required at least a two-day admission to the ICU postoperatively (MASS: 6.9% versus ST: 88%). If there truly is not a difference in these operative variables, it is possible that the potential benefit of MIS is counteracted by the more complicated nature of operating in patients with metastatic spine disease [24]. This observation was present in the study included by Payer and Sottas [30] in which mean blood loss, operative time, and complication rates were higher in patients being operated for spinal tumors versus those operated on the spine for pathology other than tumor. 

## 5. Conclusions

A systematic review of the literature yielded Class IV data suggesting that both VAST and MASS MIS modalities are efficacious means of achieving neurological improvement and alleviating pain in the treatment of metastatic spine disease. However, the magnitude of neurological improvement and/or pain alleviation cannot be accurately quantified by such retrospective studies. Such studies suggest that minimally invasive surgery for metastatic spine disease offers decreased blood loss, operative time, and complication rates in comparison to standard open spine surgery. Furthermore, these studies also suggest that MIS implementation was not limited by increased operative times. Nonetheless due to the paucity of studies and low class of available evidence, the ability to draw comprehensive conclusions is limited. Minimally invasive surgery thus remains a viable *option* for the treatment of spinal metastases. Future investigations should be conducted comparing standard surgery versus minimally invasive surgery in a prospective fashion. 

## Figures and Tables

**Table 1:** Endoscopic video-assisted thoracoscopy (VAST) outcomes. MOT: Mean operating time; LOS: Length of stay; NI: Neurological improvement rate; PA: Pain alleviation rate; CR: Complications rate; MBL: Mean blood loss.

Study	Design and procedure	Outcome results
Rosenthal et al. [20]; 1996	Retrospective analysis (*n* = 4) of outcomes associated with VAST MIS management of thoracic metastatic spine disease	MOT: 6.5 hours LOS: median 7.5 days NI: All patients experienced neurological improvement; in addition, all were independently ambulatory at time discharge and followup (mean 11 mo) PA: All patients free of pain at time of discharge and followup (mean 11 mo) CR: none MBL = mean 1450 mL

Huang et al. [24]	Retrospective analysis (*n* = 41) to analyze the complication rate in VAST MIS	MOT: 3.1 hours CR: 54% MBL: mean 775 mL

Le Huec et al. [25], 2001	Case series (*n* = 2) to report outcomes associated with the use of VAST to manage spinal metastases at the cervicothoracic junction	MOT: 2.6 hours NI: Both patients experienced neurological improvement and were independent at followup (mean 9.5 mo) PA: Both patients experienced pain relief and only one required narcotics postoperatively CR: 1 patient suffered a progressive recurrent laryngeal nerve palsy MBL: 350 mL

McLain [21], 2001	Retrospective case series (*n* = 8) to evaluate outcomes of endoscopy-assisted posterolateral approach to manage thoracic metastatic spine disease	MOT: 6 hours LOS: 6.5 days NI: All 8 patients experienced neurological improvement PA: All 8 patients experienced pain relief. Additionally 63% of patients experienced complete pain relief CR: none MBL: 1677 mL

Mobbs et al. [26], 2002	Case report (*n* = 1) of endoscope-assisted posterior decompression of a solitary renal cell carcinoma metastatic lesion	NI: Patient was neurologically intact at two-month followup. Patient initially presented with hyperreflexia PA: Patient was pain-free at two-month followup CR: Patient experienced no procedural complications

**Table 2:** Minimal access spine surgery outcomes. MOT: Mean operating time; LOS: Length of stay; NI: Neurological improvement rate; PA: Pain alleviation rate; CR: Complications rate; MBL: Mean blood loss, SVR: 2-year survival rate.

Study	Design and procedure	Outcome results
Mühlbauer et al. [27], 2000	Retrospective case series (*n* = 5) of patients undergoing lumbar corpectomy and anterior reconstruction via MASS in the setting of osteoporotic or malignancy-related compression fractures	MOT: 6 hours NI: All patients experienced neurological improvement and were ambulatory at followup (6 mo to 1 yr) PA: All patients experienced pain relief. 40% of patients did not utilize analgesics at 1-year followup CR: Segmental vessel nick via a high-speed drill. Bleeding was adequately controlled MBL: 1120 mL

Huang et al. [23], 2006	Retrospective analysis (*n* = 46) comparing MASS (*n* = 29) to standard thoracotomy (ST) (*n* = 17) in the management of thoracic spinal metastasis	MOT: MASS = 179 mins versus ST = 180 mins; *P* = .54 % Requiring 2-day ICU stay: MASS = 6.9% versus ST = 88%, *P* ≤ .001 NI: Reacquisition of ambulation postoperatively; MASS = 70.8% versus ST = 69.2%, *P* = .6 SVR: MASS = 27.4 mo versus ST = 24.8 mo, *P* = .68 CR: MASS = 24% versus ST = 29% MBL: MASS = 1,100 mL versus ST = 1,162 mL, *P* = .63

Deutsch et al. [28], 2008	Retrospective case series (*n* = 8) of patients undergoing MASS posterolateral vertebrectomy and decompression for the management of thoracic spinal metastasis	MOT: 2.2 hours LOS: 4 days NI: 62.5% of patients PA: 62.5% of patients CRs: none MBL: 227 mL

Kan and Schmidt [29], 2008	Retrospective case series (*n* = 5) of patients undergoing MASS anterior corpectomy and decompression for the management of thoracic spinal metastasis	MOT: 4.3 hours LOS: 6.25 NI: All patients experienced neurological improvement PA: All patients experienced pain alleviation CR: none MBL: 610 mL

Payer and Sottas [30], 2008	Retrospective case series (*n* = 11) analyzing operative outcomes of MASS conducted with the SynFrame (Stratec Medical, Obendorf, Switzerland) table mounted retractor in the setting of thoracic metastatic spine disease	MOT: 188 mins NI: All patients neurologically intact, at presentation remained intact and 91% of patients with preoperative deficit experienced neurological improvement CR: 18% (2/11; one dural tear and one superficial wound infection) MBL: 711 mL

Taghva et al. [31], 2010	Case report of a man undergoing vertebrectomy and expandable cage reconstruction for the management of metastatic lung adenocarcinoma localized to the thoracic spine	MOT: 7 hours LOS: 5 days NI: Patient experienced myelopathy relief and was ambulatory on postoperative day 1 PA: at 9-month followup, patient remained back pain-free with no use of analgesic medications CR: none MBL: 1200 mL

**Table 3:** Minimally invasive spine surgery outcomes summary. VAST: Video-assisted thoracoscopy; MASS: Minimal access spine surgery; mMOT: Median mean operating time; mLOS: Median mean length of stay; NI: Median neurological improvement rate; PA: Median pain alleviation rate; mCR: Median complication rate; mMBL: Median mean blood loss.

	VAST (median (range))	MASS (median (range))
*N* =	105 patients	76 patients
mMOT	4.6 hours (2.6–6.5 hours)	3.7 hours (2.2–7 hours)
mLOS	7 days (6.5–7.5 days)	5 days (4–6.25 days)
mNI:	100% (92%–100%)	95% (62.5%–100%)
mPA:	100% (94%–100%)	100% (62.5%–100%)
mCR:	0% (0%–54%)	9% (0%–24%)
mMBL	1113 mL (350–1677 mL)	905 mL (227–1200 mL)
